# Life Expectancies of South African Adults Starting Antiretroviral Treatment: Collaborative Analysis of Cohort Studies

**DOI:** 10.1371/journal.pmed.1001418

**Published:** 2013-04-09

**Authors:** Leigh F. Johnson, Joel Mossong, Rob E. Dorrington, Michael Schomaker, Christopher J. Hoffmann, Olivia Keiser, Matthew P. Fox, Robin Wood, Hans Prozesky, Janet Giddy, Daniela Belen Garone, Morna Cornell, Matthias Egger, Andrew Boulle, for the International Epidemiologic Databases to Evaluate AIDS Southern Africa (IeDEA-SA) Collaboration

**Affiliations:** 1 Centre for Infectious Disease Epidemiology and Research, University of Cape Town, Cape Town, South Africa; 2 Africa Centre for Health and Population Studies, University of KwaZulu-Natal, Mtubatuba, South Africa; 3 Centre for Actuarial Research, University of Cape Town, Cape Town, South Africa; 4 Aurum Institute, Johannesburg, South Africa; 5 Division of Infectious Diseases, Johns Hopkins School of Medicine, Baltimore, Maryland, United States of America; 6 Institute of Social and Preventive Medicine, University of Bern, Bern, Switzerland; 7 Center for Global Health and Development, Boston University, Boston, Massachusetts, United States of America; 8 Health Economics and Epidemiology Research Office, Department of Internal Medicine, School of Clinical Medicine, Faculty of Health Sciences, University of the Witwatersrand, Johannesburg, South Africa; 9 Desmond Tutu HIV Centre, University of Cape Town, Cape Town, South Africa; 10 Division of Infectious Diseases, Department of Medicine, University of Stellenbosch, Cape Town, South Africa; 11 Tygerberg Academic Hospital, Cape Town, South Africa; 12 McCord Hospital, Durban, South Africa; 13 Médecins Sans Frontières, Cape Town, South Africa; Johns Hopkins University, United States of America

## Abstract

**Background:**

Few estimates exist of the life expectancy of HIV-positive adults receiving antiretroviral treatment (ART) in low- and middle-income countries. We aimed to estimate the life expectancy of patients starting ART in South Africa and compare it with that of HIV-negative adults.

**Methods and Findings:**

Data were collected from six South African ART cohorts. Analysis was restricted to 37,740 HIV-positive adults starting ART for the first time. Estimates of mortality were obtained by linking patient records to the national population register. Relative survival models were used to estimate the excess mortality attributable to HIV by age, for different baseline CD4 categories and different durations. Non-HIV mortality was estimated using a South African demographic model. The average life expectancy of men starting ART varied between 27.6 y (95% CI: 25.2–30.2) at age 20 y and 10.1 y (95% CI: 9.3–10.8) at age 60 y, while estimates for women at the same ages were substantially higher, at 36.8 y (95% CI: 34.0–39.7) and 14.4 y (95% CI: 13.3–15.3), respectively. The life expectancy of a 20-y-old woman was 43.1 y (95% CI: 40.1–46.0) if her baseline CD4 count was ≥200 cells/µl, compared to 29.5 y (95% CI: 26.2–33.0) if her baseline CD4 count was <50 cells/µl. Life expectancies of patients with baseline CD4 counts ≥200 cells/µl were between 70% and 86% of those in HIV-negative adults of the same age and sex, and life expectancies were increased by 15%–20% in patients who had survived 2 y after starting ART. However, the analysis was limited by a lack of mortality data at longer durations.

**Conclusions:**

South African HIV-positive adults can have a near-normal life expectancy, provided that they start ART before their CD4 count drops below 200 cells/µl. These findings demonstrate that the near-normal life expectancies of HIV-positive individuals receiving ART in high-income countries can apply to low- and middle-income countries as well.

*Please see later in the article for the Editors' Summary*

## Abstract 2

Leigh Johnson and colleagues estimate the life expectancies of HIV positive South African adults who are taking antiretroviral therapy by using information from 6 programmes between 2001 and 2010.

## Editors' Summary

**Background:**

According to the latest figures, more than 34 million people worldwide currently live with HIV/AIDS. In 2011, an estimated 2.5 million people were newly infected with HIV, and in the same year 1.7 million people died from AIDS. Since the beginning of the epidemic in the 1980s, more than 60 million people have contracted HIV and nearly 30 million have died of HIV-related causes. Despite the stark statistics, the life expectancy for people infected with the AIDS virus has dramatically improved over the past decade since the introduction of an effective combination of antiretroviral drugs. In high-income countries, people who are HIV-positive can expect a near-normal life expectancy if they take these drugs (as antiretroviral treatment—ART) throughout their life.

**Why Was This Study Done?:**

Recent studies investigating the life expectancy of people living with HIV have mostly focused on the situation in high-income settings. The situation in low- and middle-income countries is vastly different. People who are diagnosed with HIV are often late in starting treatment, treatments regimes are sometimes interrupted, and a large proportion of patients are lost to follow-up. It is important to gain a realistic estimate of life expectancy in low- and middle-income countries so patients can be given the best information. So in this study the researchers used a model to estimate the life expectancy of patients starting ART in South Africa, using data from several ART programs.

**What Did the Researchers Do and Find?:**

The researchers used data collected from six programs in South Africa based in Western Cape, Gauteng, and KwaZulu-Natal between 2001 and 2010. The researchers calculated the observation time from the time of ART initiation to the date of death or to the end of the study. Then the researchers used a relative survival approach to model the excess mortality attributable to HIV, relative to non-HIV mortality rates in South Africa, over different periods from ART initiation.

Using these methods, the researchers found that over the time period, 37,740 adults started ART and 2,066 deaths were recorded in patient record systems. Of the 16,250 patients who were lost to follow-up, the researchers identified 2,947 further deaths in the population register. When they inputted these figures into their model, the researchers estimated that the mortality rate was 83.2 per 1,000 person-years of observation (PYO), and was higher in males (99.8 per 1,000 PYO) than in females (72.6 per 1,000 PYO). The researchers also found that the most significant factor determining the life expectancy of treated patients was their age at ART initiation: the average life expectancy of men starting ART varied between 27.6 years at age 20 and 10.1 years at age 60, while corresponding estimates in women were 36.8 and 14.4, respectively. Life expectancies were also significantly influenced by baseline CD4 counts; life expectancies in patients with baseline CD4 counts ≥200 cells/µl were between 70% and 86% of those of HIV-negative adults of the same age and sex, while patients starting ART with CD4 counts of <50 cells/µl had life expectancies that were between 48% and 61% of those of HIV-negative adults. Importantly, the researchers found that life expectancies were also 15%–20% higher in patients who survived their first 24 months after starting ART than in patients of the same age who had just started therapy.

**What Do These Findings Mean?:**

These findings suggest that in South Africa, patients starting ART have life expectancies around 80% of normal life expectancy, provided that they start treatment before their CD4 count drops below 200 cells/µl. Although these results are encouraging, this study highlights that health services must overcome major challenges, such as dealing with late diagnosis, low uptake of CD4 testing, loss from pre-ART care, and delayed ART initiation, if near-normal life expectancies are to be achieved for the majority of HIV-positive South Africans. With the anticipated increase in the fraction of patients starting ART at higher CD4 counts in the future, long-term survival can be expected to increase even further. It is therefore critical that appropriate funding systems and innovative ways to reduce costs are put in place, to ensure the long-term sustainability of ART delivery in low- and middle-income countries.

**Additional Information:**

Please access these websites via the online version of this summary at http://dx.doi.org/10.1371/journal.pmed.1001418.

- The International Epidemiologic Databases to Evaluate AIDS has more statistical information from world regions
- amfAR, the Foundation for AIDS Research, works with health care workers and AIDS organizations in developing countries to create and implement effective HIV research, treatment, prevention, and education strategies

## Introduction

Estimates of life expectancies of HIV-infected individuals are important in providing information to patients about their long-term prognosis, in projecting the future costs of HIV-related care, and in forecasting the likely future demographic and socioeconomic impact of HIV/AIDS [1]–[3]. Several estimates of the life expectancy of HIV-positive adults in high-income countries have been published [4]–[13], and many of these studies have shown dramatic improvements in life expectancy following the introduction of highly active antiretroviral treatment (ART). However, only one previous study has directly estimated the life expectancy of patients receiving ART in a low-income country [14]. The dearth of estimates from low- and middle-income countries is a reflection of both the later introduction of ART (with less time to accumulate long-term survival data) and the problems associated with obtaining accurate mortality estimates in these countries. High rates of loss to follow-up [15], together with high mortality in those lost to follow-up (LTFU) [16], mean that mortality is often substantially underestimated. In the absence of reliable empirical estimates, modellers have made conservative assumptions about the life expectancy of adults starting ART in low- and middle-income countries, typically around 10 y [17]–[19]. Uncertainty regarding long-term HIV mortality is frequently manifested in insurance companies' refusal of life insurance applications by HIV-positive individuals, or acceptance on very restrictive terms.

In South Africa there exists a unique opportunity to obtain accurate estimates of ART mortality in a middle-income country experiencing a generalised HIV epidemic. With around 90% of adult deaths recorded through the country's vital registration system [20]–[23], South Africa is perhaps the only African country with levels of vital registration high enough to permit independent estimation of mortality rates in patients [24]. HIV/AIDS has had a profound demographic impact in South Africa [20],[25],[26], but access to ART has expanded rapidly since 2004, with ART reaching almost 1.8 million South Africans by mid-2011 [27]. The combination of high mortality ascertainment and large patient numbers allows for greater precision in the estimation of ART mortality than is possible in most low- and middle-income countries. The objective of the present study is to estimate the life expectancy of patients starting ART in South Africa, using data from a large collaboration of ART programmes.

## Methods

### Cohort Description and Selection of Patients

The International Epidemiologic Databases to Evaluate AIDS Southern Africa (IeDEA-SA) is a collaboration of ART programmes in southern Africa [28]. All IeDEA-SA programmes obtained ethical approval from relevant local institutions before contributing anonymised patient data to this collaborative analysis. In addition, the collaboration obtained approval from the University of Cape Town Human Research Ethics Committee to receive and analyse these collaborative data. As all analyses were performed with de-identified data, most patients did not provide individual consent to participate in the study [28].

This analysis is limited to six programmes providing ART to adults in South Africa [29],[30]: the Aurum workplace treatment programme, the Aurum community treatment programme, the Hlabisa HIV treatment and care programme, the Khayelitsha HIV treatment programme, McCord Hospital, and Themba Lethu Clinic. The treatment centres are situated across three of South Africa's nine provinces (Western Cape, Gauteng, and KwaZulu-Natal), mostly in urban areas, and most of the programmes operate in the public health sector. Over the period in which patients initiated ART (March 2001 to February 2010), South African treatment guidelines in the public health sector recommended that adult ART initiation be deferred until the patient's CD4 count was below 200 cells/µl or the patient had progressed to World Health Organization clinical stage IV disease [31]. We included patients who were aged 15 y or older at the time of starting ART, were treatment-naïve, and started ART in 2001 or later. Patients were excluded from the main analysis if they had missing baseline CD4 values (no CD4 measurement between 182 d before and 14 d after starting ART) or if their baseline viral load was below 400 copies/ml (as these patients were unlikely to be ART-naïve).

### Estimation of Mortality

We calculated observation time as starting at the time of ART initiation and ending at the date of death or the date of analysis closure, whichever occurred first. Patients were considered to be LTFU if there was no record of their attendance at the clinic for at least 182 d prior to database closure and their patient records did not indicate that they had died. The date of database closure differed for each of the participating programmes, and was calculated as the last recorded visit date in each programme. The date of analysis closure was defined for each programme to be the date 182 d prior to the date of database closure, in order to allow sufficient time to determine the LTFU status of each patient.

In the case of patients who were LTFU, the approach to calculating person-years of observation (PYO) differed depending on whether the programme had recorded the patient's civil identity document (ID) number and used this information to check the vital status of the patient against the national population register. In the case of LTFU patients with ID information, we censored observation at the date of analysis closure or the date of death recorded on the population register. Inverse probability weighting was used to ensure that LTFU patients with IDs were weighted up to represent the LTFU patients without IDs; this meant assigning zero weight to the LTFU patients who had no ID (so that the censoring dates for these patients were irrelevant) and assigning weights equal to the inverse of the probability of having ID information, in those patients who had IDs. This approach has been used previously in estimating mortality rates in South African patients receiving ART [21],[32], and is analogous to the weighting methods that are commonly used to correct for non-informative censoring through double sampling [33]. Inverse probability weights were calculated by applying separate logistic regression models to the LTFU patients in each cohort, to predict the probability of having recorded ID information.

In one of the cohorts, problems with the recording of date of last clinic attendance prevented us from applying the LTFU definition and using inverse probability weighting. In this cohort, analysis was restricted to those patients with IDs, and observation time was censored at the date of death or date of analysis closure (the latter being defined as the date 30 d prior to when the national population register was last checked). In a sensitivity analysis we considered the effect of applying the same method to all of the other cohorts.

### Statistical Methods

We used a relative survival approach to model the excess mortality attributable to HIV, relative to non-HIV mortality rates in South Africa, over different durations from ART initiation [34],. The relative survival model was applied separately to male and female mortality data, allowing for four covariates: age, cohort, number of years since ART initiation, and CD4 category at ART initiation (Figure S1). HIV-associated mortality was assumed to be an exponential function of age, based on an examination of standardised mortality rates in each 5-y age interval (Figure S2). We grouped individuals into one of four baseline CD4 categories: <50, 50–99, 100–199, and ≥200 cells/µl. Mortality rates were assumed to be constant over each integer age and integer duration. We defined four duration categories: the first 12 mo after starting ART, months 13–24 after starting ART, months 25–36 after starting ART, and more than 36 mo after starting ART. The model was fitted separately for the first 12 mo and durations longer than 12 mo, to allow for differences in the effect of age, CD4 count, and cohort at different durations. A more detailed mathematical description of the model is provided in section 1 of Text S1.

We obtained estimates of non-HIV mortality, by age and sex, from an independent demographic model of the South African population [36] and used these mortality rates to calculate the life expectancy of HIV-negative individuals, for comparison purposes. The demographic model derives non-HIV mortality rates based on a modification of the Brass logit life table system, which takes into account estimates of South African mortality prior to the AIDS epidemic, changes over time in recorded numbers of deaths, and modelled estimates of trends in AIDS mortality (a more detailed explanation of the method is provided in section 2 of Text S1).

We fitted the relative survival model to the data using a maximum likelihood approach in STATA 11.0 (StataCorp), assuming that the number of deaths in each age, sex, cohort, CD4, and duration category was Poisson-distributed. Parametric bootstrapping was used to generate 1,000 alternative parameter estimates [37]. We developed a C++ programme to calculate the life table and life expectancy for various combinations of age, sex, cohort, and baseline CD4 categories. The programme was also used to estimate the fraction of patients starting ART who were expected to die from causes unrelated to HIV. We ran this programme for each of the 1,000 bootstrap-sampled parameter estimates to generate distributions of life expectancy estimates, and calculated means and 95% confidence intervals from these. Results obtained for individual cohorts were averaged for the purpose of presenting overall results. Mathematical details regarding the model fitting procedure and life expectancy calculations are included in sections 3–5 of Text S1.

### Sensitivity Analyses

To assess the sensitivity of the results to the high early mortality after ART initiation, we estimated life expectancy for patients who had survived 24 mo since ART initiation. We also limited the analysis to patients with IDs, to assess the effect of longer follow-up time (with later administrative censoring), and also to assess the effect of not applying inverse probability weighting in LTFU patients. To assess sensitivity to non-HIV mortality rates, the relative survival model was refitted after increasing the assumed non-HIV mortality rates by 50%. In addition, we refitted the model using a negative binomial model in place of the Poisson model, to assess possible bias due to over-dispersion [38]. To assess the effect of including patients with missing baseline CD4 values, we repeated the analysis after assigning CD4 values to these patients using multiple imputation [39].

Because the relative survival approach differs substantially from the more widely used abridged life table method, we also estimated life expectancy using the abridged life table approach [40],[41]. Mortality rates were calculated in each 5-y age band, stratified by sex and baseline CD4 category but not by duration. Because of the small number of patients at older ages, all observations at age 55 y and above were grouped together in a single upper age interval, consistent with the approach adopted in other abridged life table studies [9],[11],[14]. Confidence intervals were calculated using bootstrapping.

## Results

Analysis was based on 37,740 adults who started ART between March 2001 and February 2010. Table 1 shows the patient characteristics at the time of ART initiation. Relatively few patients were aged 55 y or older (3.7%), and relatively few patients had CD4 counts ≥200 cells/µl (13.2%). Following ART initiation, 2,066 deaths were recorded in patient record systems, and 16,250 patients were LTFU or were considered to have unreliable information regarding their last visit date. Of the 16,250, 13,968 had a recorded ID, and in these patients with ID, 2,947 deaths were identified in the population register. After including deaths recorded in the population register and applying the inverse probability weighting to the LTFU patients, there were 5,782 deaths during 69,514 person-years, for a mean follow-up of 1.84 y (median 1.69 y). The mortality rate was 83.2 per 1,000 PYO, and was substantially higher in males (99.8 per 1,000 PYO) than in females (72.6 per 1,000 PYO). Although mortality rates were high during the first 12 mo after starting ART, mortality rates reduced to low levels at longer durations (Figure S1 and S2). The estimated parameters of the model of HIV mortality are included in section 6 of Text S1.

**Table 1:** Patient characteristics at start of ART.

Characteristic	*n*	Percent
**Sex**		
Male	14,528	39.5%
Female	23,212	61.5%
**Age**		
15–24 y	2,697	7.1%
25–34 y	15,584	41.3%
35–44 y	12,699	33.6%
45–54 y	5,357	14.2%
55+ y	1,403	3.7%
**CD4 count**		
<50 cells/µl	10,411	27.6%
50–99 cells/µl	7,642	20.2%
100–199 cells/µl	14,689	38.9%
≥200 cells/µl	4,998	13.2%
**Year of ART initiation**		
2001–2003	913	2.4%
2004	3,079	8.2%
2005	6,122	16.2%
2006	9,683	25.7%
2007	8,630	22.9%
2008	5,956	15.8%
2009–2010	3,357	8.9%

**Figure 1:**
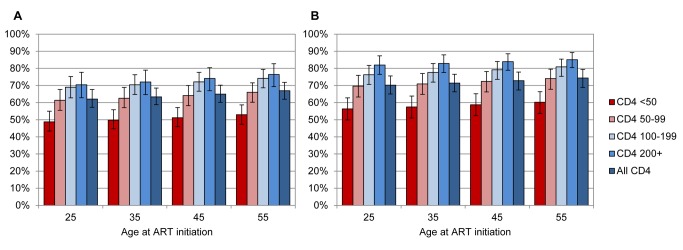
Life expectancies of patients starting ART, as proportions of life expectancies of HIV-negative adults. Proportions are plotted by age at ART initiation and baseline CD4 count, for men (A) and women (B). Bars represent means, and error bars represent 95% confidence intervals.

Table 2 summarises the model estimates of life expectancies at ART initiation. The most significant factor determining life expectancy of treated patients was age at ART initiation; the average life expectancy of men starting ART varied between 27.6 y (95% CI: 25.2–30.2) at age 20 y and 10.1 y (95% CI: 9.3–10.8) at age 60 y, while corresponding estimates in women were 36.8 (95% CI: 34.0–39.7) and 14.4 (95% CI: 13.3–15.3), respectively. Life expectancies were also significantly influenced by baseline CD4 counts; life expectancies in patients with baseline CD4 counts ≥200 cells/µl were between 70% (95% CI: 62%–77%) and 86% (95% CI: 81%–90%) of those in HIV-negative adults of the same age and sex, while patients starting ART with CD4 counts of <50 cells/µl had life expectancies that were between 48% (95% CI: 43%–55%) and 61% (95% CI: 54%–67%) of those in HIV-negative adults (Figure 1).

**Table 2:** Life expectancies (additional years of life) at ART initiation by age, sex, and baseline CD4 count.

Age at ART Initiation (in Years)	Men						Women					
	Baseline CD4 Count						Baseline CD4 Count					
	<50	50–99	100–199	200+	All^a^	Uninfected	<50	50–99	100–199	200+	All^a^	Uninfected
20	21.7 (19.2–24.5)	27.3 (24.6–30.1)	30.6 (27.8–33.5)	31.2 (27.7–34.6)	27.6 (25.2–30.2)	44.8	29.5 (26.2–33.0)	36.5 (33.1–39.9)	40.0 (36.9–43.0)	43.1 (40.1–46.0)	36.8 (34.0–39.7)	52.9
25	19.8 (17.7–22.4)	25.0 (22.6–27.5)	28.1 (25.5–30.6)	28.7 (25.6–31.6)	25.3 (23.3–27.5)	40.7	27.2 (24.1–30.4)	33.7 (30.6–36.7)	36.9 (34.1–39.5)	39.6 (37.0–42.2)	33.9 (31.5–36.5)	48.3
30	18.1 (16.2–20.3)	22.7 (20.7–25.1)	25.6 (23.4–27.8)	26.2 (23.5–28.7)	23.0 (21.3–25.0)	36.7	24.9 (22.2–27.7)	30.8 (28.1–33.5)	33.7 (31.3–36.0)	36.1 (33.7–38.4)	31.0 (28.8–33.3)	43.8
35	16.3 (14.6–18.3)	20.5 (18.7–22.6)	23.1 (21.2–25.0)	23.6 (21.2–25.9)	20.7 (19.3–22.4)	32.8	22.6 (20.1–25.1)	27.9 (25.5–30.3)	30.6 (28.3–32.6)	32.6 (30.5–34.6)	28.1 (26.1–30.1)	39.4
40	14.5 (13.1–16.2)	18.2 (16.6–19.9)	20.5 (18.9–22.1)	21.0 (18.9–22.9)	18.4 (17.1–20.0)	28.8	20.3 (18.1–22.5)	25.0 (23.0–27.1)	27.4 (25.5–29.1)	29.1 (27.3–30.8)	25.2 (23.3–27.0)	34.9
45	12.7 (11.4–14.2)	16.0 (14.5–17.4)	18.0 (16.6–19.3)	18.4 (16.7–20.0)	16.2 (15.0–17.5)	24.9	18.0 (16.1–20.0)	22.2 (20.3–24.0)	24.3 (22.6–25.8)	25.7 (24.2–27.1)	22.3 (20.7–23.9)	30.7
50	11.0 (9.9–12.3)	13.8 (12.6–15.0)	15.5 (14.4–16.7)	16.0 (14.5–17.3)	14.0 (13.0–15.1)	21.2	15.8 (14.1–17.5)	19.4 (17.8–20.9)	21.3 (19.8–22.5)	22.5 (21.2–23.6)	19.5 (18.1–20.9)	26.6
55	9.5 (8.5–10.5)	11.8 (10.8–12.8)	13.3 (12.3–14.2)	13.7 (12.4–14.8)	12.0 (11.1–12.9)	17.9	13.7 (12.2–15.1)	16.8 (15.4–18.1)	18.4 (17.1–19.4)	19.3 (18.3–20.3)	16.9 (15.6–18.0)	22.7
60	8.0 (7.1–8.9)	10.0 (9.1–10.8)	11.2 (10.3–11.9)	11.5 (10.5–12.4)	10.1 (9.3–10.8)	14.8	11.7 (10.4–12.8)	14.3 (13.1–15.3)	15.6 (14.6–16.5)	16.4 (15.5–17.2)	14.4 (13.3–15.3)	19.1

95% confidence intervals are shown in brackets.

a Standardised to the baseline CD4 distribution in Table 1.

**Figure 2:**
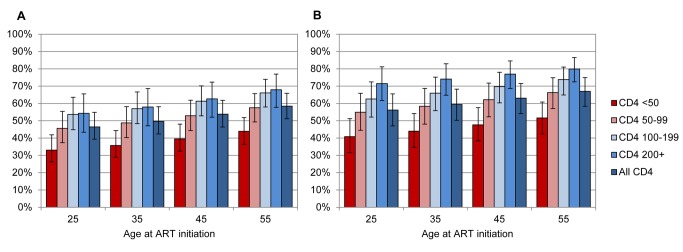
Proportion of individuals starting ART who are expected to die from causes unrelated to HIV. Proportions are plotted by age at ART initiation and baseline CD4 count, for men (A) and women (B). Bars represent means, and error bars represent 95% confidence intervals.

Figure 2 shows the estimated fraction of patients starting ART who are expected to die from causes unrelated to HIV, if non-HIV mortality rates are the same in ART patients as they are in the HIV-negative population. This fraction was higher at older ages (up to 68% [95% CI: 58%–77%] in men and 80% [95% CI: 72%–87%] in women), as the estimated mortality rates in the HIV-negative population increased more steeply with respect to age than the excess HIV mortality rates in ART patients. The fraction was also higher in women than in men, because HIV-related mortality increased more steeply with respect to age in men than in women (Table 1 of Text S1).

Life expectancies differed substantially between cohorts. For men starting ART at age 35 y, life expectancy varied between 16.6 y (95% CI: 14.4–19.2) and 28.6 y (95% CI: 26.2–30.3). Life expectancy in women starting ART at age 35 y varied between 23.6 y (95% CI: 21.4–25.9) and 35.5 y (95% CI: 33.3–36.9). The average life expectancies in Table 2 were closest to those of the public sector programmes operating in urban areas.

**Figure 3:**
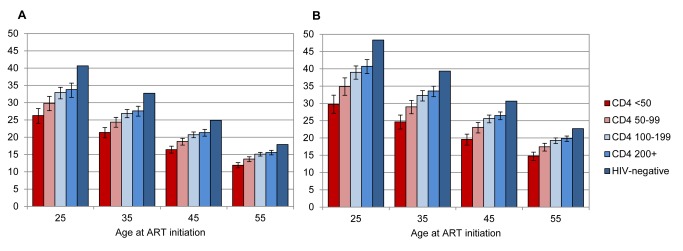
Life expectancies of patients with recorded ID, starting ART after 2006. Life expectancies are plotted by age at ART initiation and baseline CD4 count, for men (A) and women (B). Bars represent means, and error bars represent 95% confidence intervals.

In the sensitivity analysis that included only patients with recorded ID (*n* = 30,287), total PYO increased (83,199 PYO) and average follow-up time increased (2.75 y) because of the later analysis closure date. In these patients, 1,451 deaths were recorded through patient record systems, and a further 3,760 were identified through the national population register, yielding a crude mortality rate of 62.6 per 1,000 PYO. Modelled mortality rates were lower in patients starting ART after 2006 than in patients starting ART in 2006 or earlier, and life expectancies were therefore calculated separately for the two enrolment periods. Women with recorded ID who started ART in 2006 or earlier had similar life expectancies to those in the main analysis, but men with recorded ID who started ART in 2006 or earlier had life expectancies about 12% higher than those in the main analysis (Table 3). Life expectancies in patients with IDs who started ART after 2006 were in turn higher than those in patients with IDs who started ART before or in 2006 (Table 3; Figure 3). In the subset of these patients starting ART after 2006 with baseline CD4 counts ≥200 cells/µl, life expectancies were between 82% (95% CI: 77%–87%) and 88% (95% CI: 84%–91%) of those in HIV-negative individuals of the same age and sex.

**Table 3:** Sensitivity analysis of life expectancies.

		Patients with Recorded ID						
Sex and Age	Main Analysis	Started ART in 2006 or Earlier	Started ART after 2006	Patients Who Have Survived 24 mo after ART Start	Abridged Life Table Method	Multiple Imputation of Missing CD4 Counts^a^	Negative Binomial Model	Non-HIV Mortality Rates Increased by 50%
**Men**								
Age 25 y	25.3 (23.3–27.5)	28.4 (26.7–30.0)	30.6 (28.8–32.3)	30.2 (27.8–32.8)	12.0 (11.2–12.8)	25.6 (23.7–27.5)	25.2 (23.0–27.6)	24.4 (22.6–26.3)
Age 35 y	20.7 (19.3–22.4)	23.3 (22.2–24.5)	25.0 (23.7–26.1)	24.9 (23.0–26.9)	11.7 (11.1–12.4)	20.9 (19.5–22.5)	20.7 (19.1–22.5)	19.8 (18.6–21.2)
Age 45 y	16.2 (15.0–17.5)	18.1 (17.3–19.0)	19.2 (18.4–20.0)	19.5 (18.0–21.0)	10.2 (9.4–11.3)	16.3 (15.2–17.4)	16.2 (14.8–17.5)	15.1 (14.2–16.0)
Age 55 y	12.0 (11.1–12.9)	13.3 (12.7–13.9)	14.0 (13.4–14.5)	14.5 (13.4–15.5)	9.6 (8.3–11.6)	12.0 (11.2–12.8)	12.0 (11.0–12.9)	10.8 (10.2–11.3)
**Women**								
Age 25 y	33.9 (31.5–36.5)	34.3 (32.3–36.2)	35.9 (33.8–37.8)	39.1 (36.7–41.4)	15.6 (14.9–16.9)	34.0 (31.9–36.0)	33.7 (30.7–36.5)	32.3 (30.3–34.3)
Age 35 y	28.1 (26.1–30.1)	28.6 (26.8–30.0)	29.7 (28.1–31.2)	32.4 (30.6–34.3)	14.5 (13.5–16.3)	28.1 (26.5–29.6)	27.8 (25.4–30.0)	26.4 (24.9–27.8)
Age 45 y	22.3 (20.7–23.9)	22.7 (21.4–23.9)	23.6 (22.3–24.7)	25.9 (24.5–27.2)	12.8 (11.3–15.5)	22.4 (21.1–23.5)	22.1 (20.2–23.7)	20.4 (19.3–21.4)
Age 55 y	16.9 (15.6–18.0)	17.2 (16.1–18.0)	17.7 (16.7–18.5)	19.6 (18.6–20.5)	11.7 (9.0–16.3)	16.9 (16.0–17.7)	16.6 (15.2–17.7)	14.9 (14.2–15.6)

95% confidence intervals are shown in brackets. Main analysis includes all patients with CD4 measurements at time of ART initiation. Life expectancies in the main analysis are calculated from the time of ART initiation, using the relative survival method. All calculations are standardised to the baseline CD4 distribution in Table 1.

a CD4 values were imputed for 6,156 (14.0%) eligible adults starting ART.

Further sensitivity analyses are presented in Table 3. Estimates of life expectancy in patients who had survived 24 mo after starting ART were 15%–20% higher than those in patients of the same age who had just started ART. The use of the abridged life table method led to substantially lower estimates of life expectancy in patients starting ART at young ages, compared with the relative survival approach; the differences between the abridged life table and relative survival estimates were less substantial in patients starting ART at older ages. The use of multiple imputation to assign baseline CD4 values to individuals with missing information led to almost no change in life expectancy. The use of negative binomial models in place of Poisson models also produced almost identical model results. After refitting the model with 50% higher non-HIV mortality rates, life expectancy estimates were reduced by 1–2 y. Further comparison of different model estimates by CD4 strata is included in sections 7–12 of Text S1.

## Discussion

This analysis shows that South African patients starting ART have life expectancies around 80% of normal life expectancy, provided that they start treatment before their CD4 count drops below 200 cells/µl. Life expectancies are also 15%–20% higher in patients who have survived to 24 mo after starting ART than in patients of the same age who have just started therapy. Although these results are encouraging, programmes in resource-limited settings experience major challenges with late diagnosis, low uptake of CD4 testing, loss from pre-ART care, and delayed ART initiation [42]. Individuals who start ART also frequently interrupt treatment in the South African setting [43], and these interruptions are often associated with poorer immunological recovery and the development of drug resistance [44]. Health services need to overcome these challenges if near-normal life expectancies are to be achieved for the majority of HIV-positive South Africans.

South African treatment guidelines have recently changed, and it is now recommended that all HIV-infected adults should start ART when their CD4 counts fall below 350 cells/µl. Recent campaigns to increase the uptake of HIV testing [45] combined with dramatic growth in rates of ART enrolment in South Africa [27] should lead to a substantially increased proportion of patients starting ART at CD4 counts above 200 cells/µl. However, over the period to which this analysis relates, most of the patients starting ART at CD4 counts above 200 cells/µl did so because they qualified for treatment on clinical grounds. Such patients are likely to experience higher mortality than asymptomatic patients with CD4 counts above 200 cells/µl [46],[47]. These estimates of life expectancy for adults who have initiated ART with CD4 counts above 200 cells/µl may therefore be underestimates of the life expectancies in future, when a greater fraction of such individuals are likely to be asymptomatic.

A key strength of this analysis is that it incorporates data from the South African population register to obtain more accurate estimates of mortality than are usually possible in African countries. Because patients with ID information are not censored at the date of loss to follow-up, these estimates of life expectancy are inclusive of patients who are not retained in care. However, there is likely to be a small proportion of deaths (probably around 5%) that are recorded neither in the patient record system nor in the vital registration system. Although this figure is lower than the proportion of deaths missing from the vital registration system because of the added information from patient record systems, it may nevertheless lead to life expectancies of ART patients being slightly exaggerated.

Predicting future mortality for HIV-positive patients on ART is challenging. In addition to the historical bias towards enrolment of symptomatic patients, mentioned previously, our mortality estimates do not take into account potential future reductions in mortality that may occur as new drugs and salvage regimens [48] and innovations in patient management are introduced [49]. In the sensitivity analysis that was limited to patients with recorded IDs, we found higher life expectancies in patients who started ART after 2006 than in patients starting ART in or before 2006. This result may be due to a change in the disease severity of patients starting ART over time (a factor that we have not fully controlled for) or possibly improvements in regimen options and patient management.

A limitation of this analysis is the short average duration of follow-up (1.84 y), which we have addressed by controlling for differences in mortality by duration. Our method of controlling for differences by duration is based on the assumption that mortality rates are piecewise constant over 1-y duration intervals. To the extent that there is residual variation in mortality by duration, for which we have not fully controlled, our method will be biased towards overestimating mortality when the average follow-up time is short, because the observation time in each duration interval will be weighted toward the lower end of the interval, where mortality rates are likely to be highest. This bias is evident when comparing the results from the main analysis with the results from the analysis that is limited to patients with IDs; the longer average follow-up time (2.75 y) leads to slightly lower mortality rates over each duration interval (results not shown) and hence increased life expectancies. Due to the small number of patients on ART at long durations, mortality data have been aggregated for all durations greater than 36 mo. This approach may be reasonable, as average CD4 counts tend to stabilise after the first 36 mo of treatment [50],[51]. However, if HIV-related mortality continues to decline with increasing duration, the lack of data at long durations could result in some underestimation of life expectancy. On the other hand, the accumulation of drug resistance mutations at longer durations could cause long-term increases in HIV-related mortality, and mathematical modelling suggests that life expectancy may be sensitive to the number of treatment options available [52].

The principle advantage of the relative survival model, compared with the abridged life table method that is more commonly used in the estimation of life expectancy, is that it adjusts for differences in mortality by duration, which are particularly marked in resource-limited settings [53]. Although both of the measures presented here are period life expectancies rather than cohort life expectancies [54], controlling for differences in mortality by duration yields average survival times closer to those that might be expected in actual cohorts of patients initiating ART. Not controlling for duration means that the life expectancies represent the expected survival if age-specific mortality rates in the future were to remain unchanged at the high average levels measured between 2001 and 2010, which means that the abridged life table method gives too much weight to the high mortality rates soon after ART initiation. Another advantage of the relative survival approach is that it incorporates information on non-HIV mortality in the general population, which is likely to be relatively more significant at older ages. This analysis suggests that a substantial proportion of deaths in ART patients are likely to be unrelated to their HIV infection. It is therefore important that comparisons of life expectancies of HIV patients in different regions take into account differences in non-HIV mortality between settings, and the relative survival model provides a framework within which this comparison can be achieved.

Studies of life expectancies of ART patients in high-income countries that have excluded high-risk groups (people who inject drugs and patients starting ART in advanced disease) have generally estimated life expectancies that are 73%–99% of those in the general population [4],[7],[12],[55]. However, studies that have not excluded high-risk groups have estimated life expectancies that are 51%–66% of those in the general population [5],[12],[56]. These results are roughly consistent with our ratios: 62%–75% when all CD4 categories are combined, and 70%–86% when analysis is restricted to patients with baseline CD4 counts ≥200 cells/µl (Figure 1). Our ratios become even higher (87%–96%) when considering patients who started ART with CD4 counts ≥200 cells/µl and who survived their first 2 y after ART initiation.

Only one other study in Africa has estimated the life expectancy of patients starting ART [14]. This study, conducted in Uganda, estimated lower life expectancy in patients starting ART at young ages, compared with our South African estimates, but generated higher estimates than those obtained in South Africa for patients starting ART at older ages. These differences may be partly attributable to differences in methodology, as Table 3 shows that the abridged life table method (used in the Ugandan study) is likely to generate lower estimates of life expectancy at younger ages. Some of the difference may also be attributable to differences in baseline CD4 counts, which were generally higher in Uganda (the proportion with baseline CD4 count ≥100 cells/µl was 65%, compared to 52% in South Africa). Some of the difference may also be explained by differences in the approach to determining the mortality of patients LTFU, which was assumed to be 30% in the Ugandan analysis.

The generalisability of our findings—even within South Africa—is open to question. The South African cohorts participating in the IeDEA-SA Collaboration are relatively well-resourced programmes with substantial research support, mostly in urban centres. Life expectancies differed between cohorts because of differences in patients' socioeconomic characteristics as well as differences in models of ART delivery. The average results that we have presented are likely to be typical of public sector programmes in urban areas, but mortality rates may differ in rural treatment programmes and in private sector programmes. The assumption that the average non-HIV mortality rates in South Africa apply in all cohorts could also be problematic, although estimates of life expectancy did not change substantially when the model was refitted with 50% higher non-HIV mortality rates. These findings might not be typical of programmes in most other African countries, as South Africa is an upper middle-income country with rates of non-HIV mortality lower than in most other African countries [57]. HIV-related mortality in South African ART patients may also be lower than in other African countries due to virological monitoring of ART patients, which is routine in South Africa but not in most other African countries [58].

These results have important implications for the pricing models used by life insurance companies, as well as the demographic and epidemiological models that are used to forecast the impact and cost of ART programmes in low- and middle-income countries. These models have typically assumed that life expectancy after ART initiation is around 10 y [17]–[19]. Assumptions of longer life expectancy would significantly reduce the forecasts of AIDS mortality, but would also significantly increase long-term projections of numbers of patients receiving ART. With the anticipated increase in the fraction of patients starting ART at higher CD4 counts in future, long-term survival can be expected to increase even further. It is therefore critical that appropriate funding systems and innovative ways to reduce costs are put in place, to ensure the long-term sustainability of ART delivery in low- and middle-income countries.

## Supporting Information

Figure S1 **Cumulative survival after ART initiation, compared to age-standardised survival rates in the HIV-negative population.** HIV-positive survival curves are calculated by grouping all ages and cohorts together and applying Kaplan-Meier methods to calculate proportions surviving, after including data from the national population register and applying inverse probability weighting. HIV-negative survival curves are calculated for a hypothetical cohort with the same initial age distribution as the HIV-positive cohort.
(PDF)

Figure S2 **Annual mortality rates stratified by age, sex, and time since ART initiation.** Dots represent observed mortality rates, standardised to the CD4 distribution in Table 1 and calculated over 5-y age groups. Vertical lines represent 95% confidence intervals.
(PDF)

Text S1 **Technical appendix.**
(PDF)

## Abbreviations

ART: antiretroviral treatment
ID: identity document
IeDEA-SA: International Epidemiologic Databases to Evaluate AIDS Southern Africa
LTFU: lost to follow-up
PYO: person-years of observation
