# Supplementary material for: Life Expectancies of South African Adults Starting Antiretroviral Treatment: Collaborative Analysis of Cohort Studies
Source: PLoS Med. 2013 Apr 9;10(4):e1001418. doi: 10.1371/journal.pmed.1001418 (PMC3621664; doi:10.1371/journal.pmed.1001418)
Supplement: Figure S1 — Cumulative survival after ART initiation, compared to age-standardised survival rates in the HIV-negative population. HIV-positive survival curves are calculated by grouping all ages and cohorts together and applying Kaplan-Meier methods to calculate proportions surviving, after including data from the national population register and applying inverse probability weighting. HIV-negative survival curves are calculated for a hypothetical cohort with the same initial age distribution as the HIV-positive cohort. (PDF) [file pmed.1001418.s001.pdf]

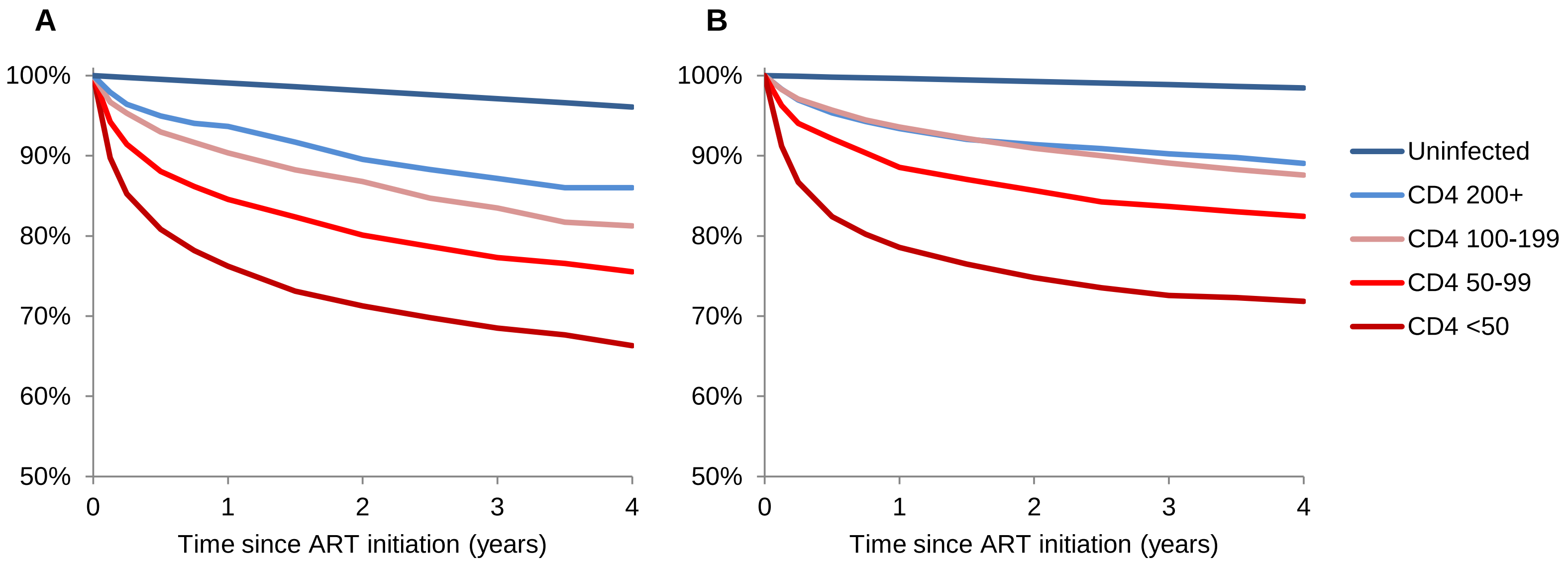

Figure S1: Cumulative survival after ART initiation, compared to age-standardized survival rates in the HIV-negative population, in males (A) and females (B). HIV-positive survival curves are calculated by grouping all ages and cohorts together and applying Kaplan-Meier methods to calculate proportions surviving, after including data from the national population register and applying inverse probability weighting. HIV-negative survival curves are calculated for a hypothetical cohort with the same initial age distribution as the HIV-positive cohort.
