# Supplementary material for: Life Expectancies of South African Adults Starting Antiretroviral Treatment: Collaborative Analysis of Cohort Studies
Source: PLoS Med. 2013 Apr 9;10(4):e1001418. doi: 10.1371/journal.pmed.1001418 (PMC3621664; doi:10.1371/journal.pmed.1001418)
Supplement: Figure S2 — Annual mortality rates stratified by age, sex, and time since ART initiation. Dots represent observed mortality rates, standardised to the CD4 distribution in Table 1 and calculated over 5-y age groups. Vertical lines represent 95% confidence intervals. (PDF) [file pmed.1001418.s002.pdf]

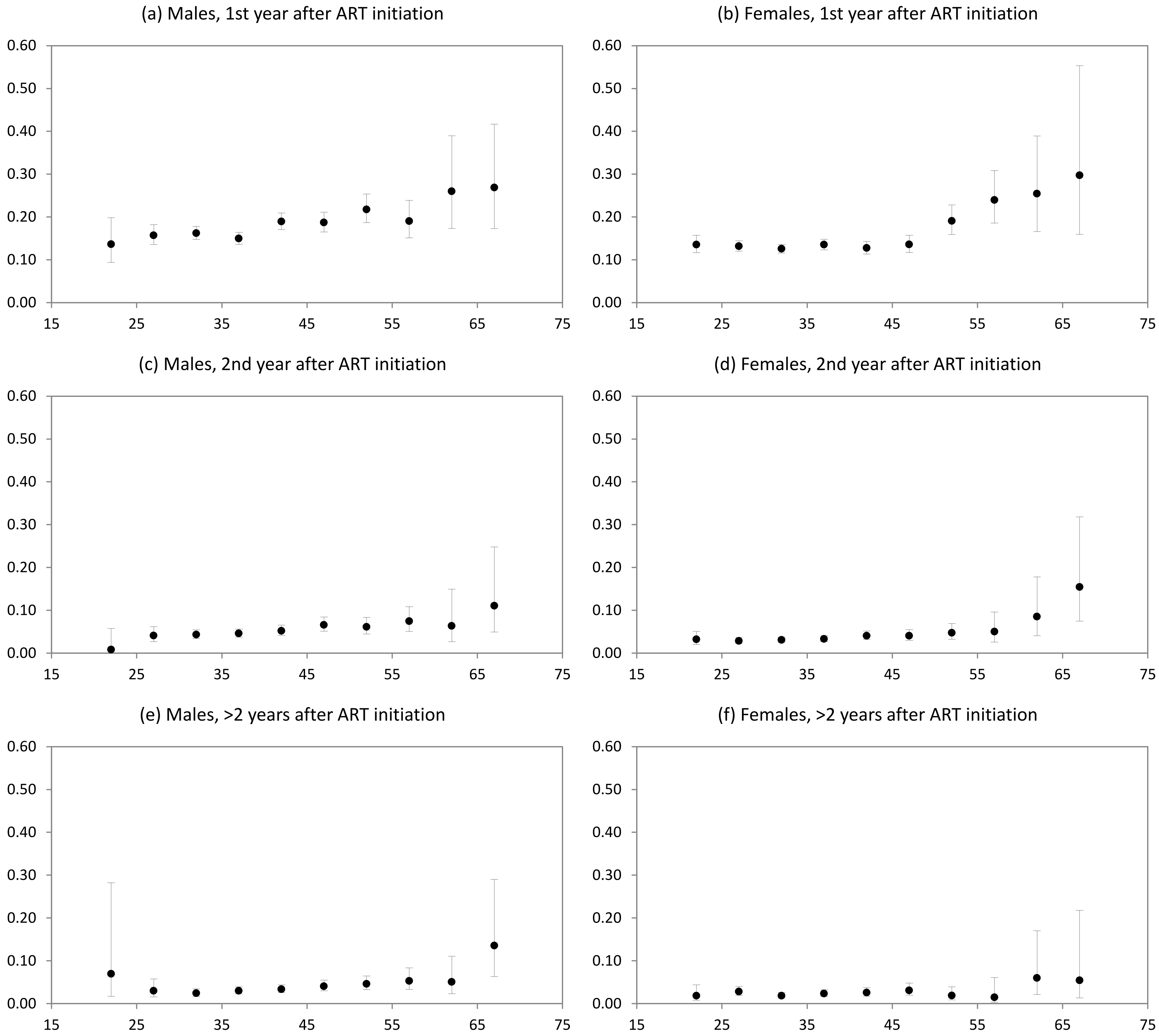

Figure S2: Annual mortality rates stratified by age, sex and time since ART initiation. Dots represent observed mortality rates, standardized to the CD4 distribution in Table 1 and calculated over 5-year age groups. Vertical lines represent 95% confidence intervals.
