# Supplementary material for: Life Expectancies of South African Adults Starting Antiretroviral Treatment: Collaborative Analysis of Cohort Studies
Source: PLoS Med. 2013 Apr 9;10(4):e1001418. doi: 10.1371/journal.pmed.1001418 (PMC3621664; doi:10.1371/journal.pmed.1001418)
Supplement: Text S1 — Technical appendix. (PDF) [file pmed.1001418.s003.pdf]

Technical appendix

Table of Contents

1. Relative survival model 2

2. Estimation of non-HIV mortality 3

3. Calculation of life expectancies 5

4. Comparison with mortality in uninfected individuals 6

5. Calculation of likelihood function 6

6. Relative survival model parameter estimates 7

7. Comparison with abridged life table method 10

8. Comparison with estimates obtained using imputation 12

9. Comparison with estimates obtained when excluding patients without ID 13

10. Comparison with life expectancy estimates 24 months after starting ART 17

11. Comparison with alternative non-HIV mortality assumptions 17

12. Comparison with negative binomial model 18

References 20

1. Relative survival model

Relative survival models evaluate the excess mortality attributable to a particular disease, in a cohort of patients with the disease, when compared to the mortality in a comparable group of individuals in the general population who do not have the disease [1,2]. The mortality rate in a patient with certain characteristics (represented by covariate vector **z**) can be expressed as

$$\mu(\mathbf{z}) = \mu^0(\mathbf{z}) + \exp(\mathbf{z\beta}),$$

where $\mu^0(\mathbf{z})$ is the mortality rate that would be expected in an individual with the same characteristics who does not have the disease, and $\boldsymbol{\beta}$ is a vector of coefficients reflecting the effect of each of the covariates on the disease-specific mortality. As noted by Dickman *et al* [1], these mortality rates are usually modelled as piecewise constant hazards over different intervals of follow-up time, typically of 12-month length. The advantage of this approach is that it is simple to relax the assumption of proportional hazards if the hazards in different covariate strata do not remain proportional over follow-up. The relative survival approach has previously been used by van Sighem *et al* [3,4] in estimating life expectancies of HIV-diagnosed adults in the Netherlands, by Bhaskaran *et al* [5] in assessing mortality trends in the CASCADE Collaboration, and by Brinkhof *et al* [6] in estimating mortality after ART initiation in African cohorts.

In the current analysis, we apply the relative survival model separately to male and female mortality data, allowing for four covariates: age at ART initiation (x), number of complete years since ART initiation (d), CD4 category at ART initiation (i) and cohort (j). Individuals are grouped into one of four baseline CD4 categories: CD4 < 50 cells/µl (i = 0), CD4 50-99 cells/µl (i = 1), CD4 100-199 cells/µl (i = 2) and CD4 of 200 cells/µl or higher (i = 3). Mathematically, the model of mortality can be expressed as follows:

$$\mu_{x,d}(i, j) = \mu^0_{x+d} + \exp(\gamma_d + \eta_d \times (x + d) + g_d(i) + \theta_d(j)), \qquad (1)$$

where μ^0_{x+d} is the mortality rate that would be expected in HIV-negative individuals, γ_d is the excess HIV mortality at duration d, η_d is the increase in HIV mortality per year of age, $g_d(i)$ is the difference in HIV-specific mortality between individuals in baseline CD4 category i and individuals with baseline CD4 counts < 50 cells/µl, and $\theta_d(j)$ is the difference in HIV mortality between individuals in cohort j and individuals in cohort 4 (all of the HIV mortality parameters are defined on the natural log scale). Mortality is assumed to be constant over different integer ages and integer durations. The CD4 effect, cohort effect and age effect all are assumed to depend on duration, with the model being fitted separately for the first 12 months after ART initiation and durations greater than 12 months. The rationale for fitting different baseline CD4 effects in different duration categories is that one would expect baseline CD4 to be more predictive of mortality during the first few months after starting ART than at longer durations [7], when factors affecting the extent of the CD4

response become relatively more significant. The effect of age might also be expected to vary in relation to treatment duration: older adults tend to have greater short-term virological suppression than younger adults [8-13], but their long-term CD4 recovery is less substantial than that in younger adults [11-14]. We also anticipated that the cohort effect on mortality at early durations might differ substantially from that at later durations because the programmatic factors that influence mortality at early durations are different from those that influence mortality at later durations [10,15].

The duration parameters are estimated separately for each of four intervals: the first 12 months after starting ART (d = 0), months 13-24 after starting ART (d = 1), months 25-36 after starting ART (d = 2) and durations of more than 36 months (d = 3). This means that for $d \geq 3$,

$$\mu_{x,d+1}(i, j) = \mu_{x+1,d}(i, j). \qquad (2)$$

Over each of these four intervals, the mortality rate is assumed to be constant with respect to duration, though the mortality rate can change in relation to age. The assumption of a constant mortality rate is not realistic at short durations, but for the purpose of calculating life expectancies it is the cumulative survival probability over longer durations that is most important, and changing the interval definitions at short durations (for example, to 0-6 months in the first interval) does not change the life expectancy substantially. Increasing the lower limit on the upper duration interval (for example, to more than 48 months) could have a more material impact, although we did not find a statistically significant improvement in the model fit to the data if we included this additional duration category.

2. Estimation of non-HIV mortality

Due to the lack of vital registration systems in many developing countries [16], direct estimates of mortality by age and sex are often not available. To address this problem, life tables are often estimated for developing countries by taking 'standard' life tables (which define mortality rates by individual age and sex) and adjusting these to be consistent with aggregate measures of mortality (such as the under-5 mortality rate or probability of death between ages 15 and 60), as estimated in local surveys [17]. For example, in the Brass logit life table system [18], the proportion of individuals who survive to age x (l_x) is modelled using the equation

$$\frac{1}{2}\ln\left(\frac{1-l_x}{l_x}\right) = \alpha + \frac{1}{2}\beta\ln\left(\frac{1-l_x^S}{l_x^S}\right),$$

where l_x^S is the proportion of individuals who survive to age x in the standard life table. In this model there are only two parameters that need to be estimated in adjusting the standard life table to the local population: α and β (the former determines the adjustment to the overall level of mortality, the latter determines the adjustment to the age gradient in mortality rates).

In South Africa and other countries facing generalized HIV/AIDS epidemics, the use of these simple adjustments to standard life tables has become problematic because of the dramatic

change in the patterns of mortality (by age and sex) caused by AIDS. One possible solution to this problem is to split all-cause mortality into HIV mortality and non-HIV mortality, the former being estimated by mathematical models that are fitted to local HIV prevalence data, and the latter being estimated through the adjustment of standard life tables (on the assumption that AIDS would not substantially change the age pattern of non-HIV mortality). This is the process that has been followed in the estimation of mortality rates in the ASSA2008 AIDS and Demographic model, published by the Actuarial Society of South Africa (ASSA) [19]. The procedure followed is to

a) use estimates of mortality in 1985 [20], when HIV prevalence in the South African population was negligible [21,22], as the standard in the estimation of non-HIV mortality;
b) use the time-dependent Brass logit life table approach to define non-HIV mortality in each subsequent year;
c) use ASSA2008 estimates of HIV-related mortality in each year (based on calibration to South African HIV prevalence data from antenatal clinic surveys and household surveys); and
d) vary the α and β parameters and model HIV parameters, through an iterative process, until the combined model estimates of all-cause mortality (by age and sex) are consistent with the numbers of deaths in each year, as recorded in the National Population Register [23], after correcting for under-reporting of deaths.

An obvious limitation of this method is that it involves the estimation of non-HIV mortality without any direct data on the cause of death. To the extent that there is uncertainty in the model estimates of AIDS mortality, this will lead to uncertainty in estimates of non-HIV mortality. The ASSA2008 model makes several assumptions about age distributions of sexual activity, rates of HIV mortality in untreated HIV-infected individuals and levels of bias in HIV prevalence surveys, all of which influence the level and age distribution of HIV-related mortality. These assumptions are subject to significant uncertainty [24], which prevents the precise estimation of non-HIV mortality. However, it is important to note that because of the very distinct change in the pattern of deaths by age and sex, brought about by AIDS, the age- and sex-specific death data over time are actually very informative regarding the extent of AIDS mortality, even without cause-of-death data. It is also worth noting that two independent assessments, based on cause-of-death data, have validated the estimates of AIDS mortality produced by a previous version of the ASSA model [25,26]. Although the non-AIDS mortality estimates cannot be quantified with pinpoint accuracy, they are likely to be roughly of the right order of magnitude.

In the present analysis, we have used the ASSA2008 lite model estimates of non-HIV mortality in 2005. Although other years could have been chosen, 2005 was selected because it was close to the median date of ART enrolment in the IeDEA-SA collaboration [27], and because ART coverage in South Africa in 2005 was still relatively low [28], so that potential bias due to mis-specification of mortality on ART would not have been substantial. The ASSA2008 estimates of non-HIV mortality do not change substantially by year; for example, annual non-HIV mortality probabilities in 40-year old men drop steadily from 0.0079 in 2000 to 0.0074 in 2007, and corresponding probabilities in 40-year old women drop from 0.0041 in 2000 to 0.0037 in 2007. Estimates of non-HIV mortality would therefore not change substantially if alternative years were selected.

3. Calculation of life expectancies

We define $l_{x,d}(i,j)$ to be the proportion of individuals starting ART at exact age *x*, in CD4 category *i* and cohort *j*, who survive for *d* years ($l_{x,0}(i,j)=1$). The proportion of individuals who survive for 1 year is

$$l_{x,1}(i,j)=l_{x,0}(i,j)\times\exp\left(-\mu_{x,0}(i,j)\right).$$

Similarly, the proportion who survive for 2 years is

$$l_{x,2}(i,j)=l_{x,1}(i,j)\times\exp\left(-\mu_{x,1}(i,j)\right),$$

and the proportion who survive for *d* years (where *d* is an integer > 3) is

$$\begin{aligned} l_{x,d}(i,j) &= l_{x,d-1}(i,j)\times\exp\left(-\mu_{x,d-1}(i,j)\right) \\ &= l_{x,d-1}(i,j)\times\exp\left(-\mu_{x+d-4,3}(i,j)\right), \end{aligned}$$

the latter result following from equation (2). Although we have defined $l_{x,d}(i,j)$ only for integer values of *d*, it can be considered a continuous function of *d*. The life expectancy of an individual starting ART at age *x*, in CD4 category *i* and cohort *j*, is then defined as

$$\begin{aligned} e_x(i,j) &= \int_0^\infty l_{x,t}(i,j)dt \\ &\approx \left\{\sum_{d=0}^{100-x} 0.5\left(l_{x,d}(i,j)+l_{x,d+1}(i,j)\right)\right\}+l_{x,101-x}(i,j)\,e_{99,2}(i,j), \end{aligned}$$

where $e_{99,2}(i,j)\approx 1/\mu_{99,2}(i,j)$. As there are very few individuals who would be expected to survive to age 101, the use of the approximation to the life expectancy at age 101 (which effectively assumes constant mortality after age 101) has negligible effect on the accuracy of $e_x(i,j)$, provided *x* << 100. The life expectancy of an individual who started ART at age *x* and has survived for 2 years is similarly calculated as

$$e_{x,2}(i,j)\approx\frac{\sum_{d=2}^{100-x} 0.5\left(l_{x,d}(i,j)+l_{x,d+1}(i,j)\right)}{l_{x,2}(i,j)}+\frac{l_{x,101-x}(i,j)}{l_{x,2}(i,j)}e_{99,2}(i,j),$$

for *x* < 99.

4. Comparison with mortality in uninfected individuals

The life expectancies that are calculated using the relative survival model are compared with the life expectancies that would be expected in HIV-negative South Africans of the same age and sex. Because these HIV-negative life expectancies are calculated using mortality rates in HIV-negative individuals, they represent the life expectancies of individuals who *remain* HIV-negative in future, and are therefore higher than the life expectancies of currently HIV-negative individuals who may become infected with HIV in future (this difference may be substantial due to the high lifetime risk of HIV in many African countries [24,29-31]).

The non-HIV mortality rates are also used in calculating the proportion of patients who are expected to die from non-HIV causes. The probability that death is unrelated to HIV, for an individual starting ART at exact age x, in CD4 category i and cohort j, is

$$\left\{\sum_{d=0}^{100-x}\left(l_{x,d}(i,j)-l_{x,d+1}(i,j)\right)\times\frac{\mu_{x+d}^{0}}{\mu_{x,d}(i,j)}\right\}+l_{x,101-x}(i,j)\times\frac{\mu_{101}^{0}}{\mu_{99,2}(i,j)}. \qquad (3)$$

5. Calculation of likelihood function

For individuals in CD4 category i and cohort j at the time of ART initiation, aged x in the first 12 months after starting ART, we define $Y_{x,0}(i,j)$ to be the person-years of observation and $R_{x,0}(i,j)$ to be the number of deaths during the first 12 months after starting ART. Observation time is censored upon turning age $x + 1$ and deaths exclude those that occur after turning age $x + 1$. Observation time includes individuals who turned age x during their first year after starting ART, with observation time starting from when they turned x. It is assumed that deaths are Poisson-distributed, with the expected number of deaths being

$$Y_{x,0}(i,j)\times\mu_{x,0}(i,j),$$

where $\mu_{x,0}(i,j)$ is the mortality rate predicted by the relative survival model in equation (1). The likelihood of observing $R_{x,0}(i)$ deaths is thus

$$L_{x,0}(i,j)=\frac{\exp\left(-Y_{x,0}(i,j)\mu_{x,0}(i,j)\right)\left(Y_{x,0}(i,j)\mu_{x,0}(i,j)\right)^{R_{x,0}(i,j)}}{R_{x,0}(i,j)!}.$$

The log of the likelihood, for all ages, cohorts and CD4 categories, is

$$\begin{aligned}\log(L_0)&=\log\left(\prod_{i=0}^{3}\prod_{j=1}^{6}\prod_{x}L_{x,0}(i,j)\right)\\&=\sum_{i=0}^{3}\sum_{j=1}^{6}\sum_{x}\left[-Y_{x,0}(i,j)\mu_{x,0}(i,j)\right.\\&\qquad\left.+R_{x,0}(i,j)\log\left(Y_{x,0}(i,j)\mu_{x,0}(i,j)\right)-\log\left(R_{x,0}(i,j)!\right)\right]\end{aligned}. \qquad (4)$$

The likelihood L_0 can be considered a function of ten parameters from equation (1): γ_0, η_0, $g_0(1)$, $g_0(2)$, $g_0(3)$, $\theta_0(1)$, $\theta_0(2)$, $\theta_0(3)$, $\theta_0(5)$ and $\theta_0(6)$.

Similarly, for individuals aged x in the second 12 months after starting ART, in CD4 category i and cohort j at the time of ART initiation, we define $Y_{x-1,1}(i, j)$ to be the number of person-years of observation and $R_{x-1,1}(i, j)$ to be the number of deaths, during the second 12 months after starting ART. The log likelihood for the second 12 months after starting ART, $\log(L_1)$, is calculated using a formula similar to that in equation (4), and is a function of the parameters γ_1, η_1, $g_1(1)$, $g_1(2)$, $g_1(3)$, $\theta_1(1)$, $\theta_1(2)$, $\theta_1(3)$, $\theta_1(5)$ and $\theta_1(6)$.

The log likelihood is similarly defined for durations 25-36 months and >36 months. However, in the interests of parsimony, the η, g and θ parameters are assumed to be the same as those over the 13-24 month duration, so that the only additional parameters specific to the later durations are γ_2 and γ_3. This means that one maximum likelihood model is fitted for all durations greater than 12 months.

Although we have not included subscripts to indicate sex, all analyses are conducted separately for males and females. There are thus four separate models fitted to the IeDEA Southern Africa data (for 0-12 and >12 month duration categories and for males and females separately). The $Y_{x-d,d}(i, j)$ and $R_{x-d,d}(i, j)$ values are generated for each of the four models using STATA version 11.0 (StataCorp, College Station, TX, USA).

6. Relative survival model parameter estimates

Relative survival model estimates for males and females, over each of the two duration categories, are shown in Table 1, together with average mortality rates. Mortality rates were substantially higher during the first 12 months after starting ART than over subsequent durations. Excess HIV mortality increased in relation to age in all four analyses, and was significantly lower in patients starting ART at higher CD4 counts. Excess HIV mortality also differed significantly between cohorts.

To assess whether the number of duration categories was appropriate, we reran the model replacing the upper duration category (>36 months) with two categories (37-48 months and >48 months). This did not significantly improve the fit of the model, either in males ($p = 0.33$) or females ($p = 0.22$), and the γ_d parameters in the >48 month category were no lower than those in the 36-48 month category. We also reran the model to assess whether a more parsimonious model with only three duration categories might be more appropriate; for this analysis we combined the 25-36 and >36 month categories into a single category. However, we found that the model with four duration categories provided a substantially better fit to the data, both in men ($p = 0.061$) and women ($p = 0.003$).

In selecting the final model, we also considered whether year of ART initiation might be a significant explanatory variable. The cohort was separated into individuals who started ART up to 2005 and individuals who started ART after 2005. The effect of starting ART after 2005 did not prove to have a significant effect on male mortality during the first year after

ART initiation (HR 0.99, p = 0.80), female mortality during the first year after ART initiation (HR 1.01, p = 0.81), male mortality after the first year (HR 0.89, p = 0.35) or female mortality after the first year (HR 1.06, p = 0.64). As a result, we did not include year of ART initiation as a variable in our final model.

Table 1: Mortality rates and model estimates

	Symbol	Males, by time since ART start		Females, by time since ART start	
		<12 month	>12 months	<12 month	>12 months
Number of deaths		2,020	668	2,436	658
Person years		11,575	15,350	19,005	23,584
Crude mortality rate per PYO		0.1745	0.0435	0.1282	0.0279
Excess HIV mortality	$\exp(\gamma_d)$	0.155 (0.120-0.199)	0.036 (0.021-0.062)	0.166 (0.137-0.202)	0.028 (0.018-0.044)
Relative HIV mortality at					
Durations 25-36 months	$\exp(\gamma_2 - \gamma_1)$	-	0.63 (0.50-0.80)	-	0.62 (0.50-0.77)
Durations >36 months	$\exp(\gamma_3 - \gamma_1)$	-	0.44 (0.31-0.63)	-	0.35 (0.24-0.52)
Increase in HIV mortality for each 10-year increase in age	$\exp(10\eta_d)$	1.08 (1.02-1.14)	1.13 (1.01-1.28)	1.07 (1.02-1.12)	1.09 (0.98-1.22)
Hazard ratio relative to CD4 <50					
Baseline CD4 50-99	$\exp(g_d(1))$	0.56 (0.50-0.63)	0.68 (0.52-0.89)	0.46 (0.42-0.52)	0.69 (0.54-0.87)
Baseline CD4 100-199	$\exp(g_d(2))$	0.31 (0.27-0.35)	0.55 (0.43-0.70)	0.24 (0.22-0.27)	0.56 (0.45-0.69)
Baseline CD4 200+	$\exp(g_d(3))$	0.18 (0.15-0.23)	0.57 (0.41-0.80)	0.23 (0.19-0.27)	0.37 (0.26-0.53)
Hazard ratio relative to cohort 4					
Cohort 1	$\exp(\theta_d(1))$	1.51 (1.25-1.81)	1.55 (1.07-2.24)	1.24 (1.08-1.43)	1.28 (0.93-1.76)
Cohort 2	$\exp(\theta_d(2))$	2.25 (1.90-2.67)	1.00 (0.70-1.42)	2.14 (1.89-2.43)	1.43 (1.08-1.90)
Cohort 3	$\exp(\theta_d(3))$	1.44 (1.17-1.76)	1.02 (0.70-1.50)	0.89 (0.54-1.46)	1.19 (0.52-2.71)
Cohort 5	$\exp(\theta_d(5))$	1.23 (1.03-1.48)	0.90 (0.63-1.31)	1.01 (0.88-1.16)	1.20 (0.90-1.60)
Cohort 6	$\exp(\theta_d(6))$	0.72 (0.55-0.95)	0.13 (0.03-0.63)	0.62 (0.49-0.79)	0.18 (0.06-0.58)

95% confidence intervals are shown in brackets. PYO = person-year of observation.

7. Comparison with abridged life table method

Table 2 compares estimates of life expectancy by age, sex and baseline CD4 count, calculated using three different methods. The relative survival model (RS) is the model described previously and in the main text. Two approaches to applying the abridged life table method are considered: an approach in which means and standard errors are calculated using parametric bootstrapping (with 1000 replications), and the more widely-used approach, developed by Chiang, in which Taylor series are used to approximate the standard errors [32,33].

The relative survival approach yields higher estimates of life expectancy than the abridged life table approaches at young ages, and the extent of this difference is greatest in patients starting ART at low CD4 counts. At older ages, in patients starting ART with CD4 ≥200 cells/μl, the abridged life table methods yield slightly higher estimates of life expectancy than the relative survival approach. The abridged life table approach does not take into consideration differences in mortality rates by duration, and recently-enrolled individuals who are at a high mortality risk are therefore over-represented if there is a short average follow-up time. The differences in mortality by duration are greatest in individuals who start ART at very low CD4 counts (Table 1), and the extent of the bias in estimating life expectancy using abridged life tables is therefore greatest in low CD4 categories.

The narrowing of the difference between the relative survival and abridged life table estimates at the older ages is likely to be because of the assumption, made in the abridged life table method, that mortality rates are constant in the upper age interval. If mortality rates are actually increasing in relation to age over the upper age interval (55+ in this analysis), then estimating a constant mortality rate based on individuals who tend to be at the lower end of the interval can lead to significant over-estimation of the life expectancy in the upper age interval. This bias offsets the bias described in the previous paragraph, so that the abridged life table method under-estimates life expectancy by less at older ages than at younger ages, and might even over-estimate life expectancy.

Although average estimates of life expectancy are similar when comparing the abridged life table method with bootstrapping (ALB) and the abridged life table method developed by Chiang (ALC), the Chiang method yields substantially lower standard error estimates, particularly for patients starting ART at older ages. This is likely to be because the Chiang method ignores uncertainty regarding the mortality rate in the upper age interval. This would not be a major source of discrepancy if relatively few patients survived to the upper age interval, but because the lower limit on the upper age interval is relatively low in this analysis (age 55), ignoring the uncertainty in the upper age interval leads to substantial exaggeration of precision. This exaggeration of precision is greatest for those patients starting ART who have the greatest probability of surviving to age 55, i.e. older patients and patients starting ART with high CD4 counts. The relatively small number of deaths in women aged 55+ with baseline CD4 ≥200 cells/μl leads to substantial instability when using the abridged life table method with bootstrapping, making comparison with the other methods difficult for older women with high baseline CD4 counts.

Table 2: Comparison of life expectancy estimates from the relative survival model (RS), abridged life table method with bootstrapping (ALB) and abridged life table method developed by Chiang (ALC)

	Baseline CD4 <50			Baseline CD4 50-99			Baseline CD4 100-199			Baseline CD4 200+		
	RS	ALB	ALC	RS	ALB	ALC	RS	ALB	ALC	RS	ALB	ALC
Men												
Age 25	19.8	5.9	5.9	25.0	8.8	8.8	28.1	14.7	14.7	28.7	21.4	20.7
	(1.20)	(0.34)	(0.31)	(1.30)	(0.76)	(0.70)	(1.25)	(0.74)	(0.71)	(1.56)	(1.72)	(0.68)
Age 35	16.3	6.5	6.4	20.5	10.0	10.0	23.1	13.8	13.7	23.6	18.8	18.3
	(0.92)	(0.23)	(0.21)	(0.99)	(0.49)	(0.42)	(0.95)	(0.54)	(0.44)	(1.19)	(1.81)	(0.42)
Age 45	12.7	5.7	5.7	16.0	8.5	8.4	18.0	11.8	11.8	18.4	17.4	16.6
	(0.71)	(0.33)	(0.24)	(0.74)	(0.69)	(0.45)	(0.70)	(0.72)	(0.43)	(0.88)	(2.59)	(0.39)
Age 55	9.5	5.2	5.1	11.8	9.6	9.5	13.3	10.0	9.9	13.7	17.9	16.4
	(0.53)	(0.69)	(-)	(0.53)	(1.63)	(-)	(0.50)	(1.19)	(-)	(0.61)	(4.31)	(-)
Women												
Age 25	27.2	7.0	7.0	33.7	13.7	13.7	36.9	20.1	20.1	39.6	23.4	21.4
	(1.58)	(0.25)	(0.25)	(1.53)	(0.59)	(0.52)	(1.39)	(0.70)	(0.49)	(1.32)	(4.19)	(0.46)
Age 35	22.6	7.8	7.7	27.9	13.1	13.0	30.6	17.6	17.5	32.6	21.8	18.9
	(1.25)	(0.29)	(0.27)	(1.20)	(0.79)	(0.53)	(1.09)	(0.89)	(0.49)	(1.02)	(5.93)	(0.45)
Age 45	18.0	6.9	6.8	22.2	12.4	12.1	24.3	14.5	14.3	25.7	20.9	16.5
	(0.97)	(0.41)	(0.34)	(0.91)	(1.31)	(0.62)	(0.82)	(1.25)	(0.52)	(0.76)	(9.01)	(0.45)
Age 55	13.7	4.4	4.3	16.8	10.5	10.1	18.4	13.1	12.9	19.3	24.0	16.2
	(0.72)	(0.66)	(-)	(0.66)	(2.34)	(-)	(0.59)	(2.09)	(-)	(0.53)	(16.1)	(-)

Standard errors are shown in brackets.

In summary, the abridged life table method has three potential limitations when it is used to estimate life expectancies of HIV-positive patients starting ART. Firstly, the method does not take into account differences in mortality by duration, and this is likely to lead to under-estimation of life expectancy if average follow-up time is short, especially in patients starting ART at low CD4 counts. Secondly, the method assumes a constant rate of mortality in the upper age interval, which is likely to lead to the life expectancy in the upper age interval (and at older ages) being over-estimated. Thirdly, the method recommended by Chiang for calculating standard errors tends to exaggerate the precision associated with the life expectancy, particularly at older ages.

8. Comparison with estimates obtained using imputation

For 6,156 (14.0%) of the eligible adults starting ART, no CD4 measurement was available in the period between 182 days before and 14 days after starting ART. Multiple imputation by chained equations [34] was used to assign baseline CD4 values to these patients with missing CD4 values. Imputation was conducted using the ICE command in Stata 11.0 (StataCorp, College Station, TX, USA), with five imputations. The observed CD4 values were transformed using a square root transformation before the imputation was conducted, in order to achieve a more ‘normal’ distribution of baseline CD4 values. The imputation model included age, sex, CD4 count (on square root scale), year, cohort, outcome, time to outcome and inverse probability weight.

Life expectancies calculated using multiple imputation (MI) are compared with those calculated after excluding patients with missing baseline CD4 counts (EX), in Table 3. In all age and baseline CD4 strata, life expectancies estimated using multiple imputation of missing baseline CD4 values are very similar to those obtained in the main analysis, and standard error estimates tend to be lower when missing baseline CD4 values are imputed.

Table 3: Comparison of life expectancy estimates obtained when excluding patients with missing baseline CD4 counts (EX) and when using multiple imputation (MI) to assign baseline CD4 values to patients with missing information

	CD4 <50		CD4 50-99		CD4 100-199		CD4 200+	
	EX	MI	EX	MI	EX	MI	EX	MI
Men								
Age 25	19.8	19.8	25.0	25.2	28.1	28.4	28.7	29.9
	(1.20)	(1.08)	(1.30)	(1.28)	(1.25)	(1.11)	(1.56)	(1.39)
Age 35	16.3	16.2	20.5	20.6	23.1	23.2	23.6	24.5
	(0.92)	(0.82)	(0.99)	(0.97)	(0.95)	(0.85)	(1.19)	(1.05)
Age 45	12.7	12.6	16.0	16.0	18.0	18.0	18.4	19.0
	(0.71)	(0.63)	(0.74)	(0.71)	(0.70)	(0.63)	(0.88)	(0.76)
Age 55	9.5	9.4	11.8	11.8	13.3	13.3	13.7	14.0
	(0.53)	(0.48)	(0.53)	(0.51)	(0.50)	(0.45)	(0.61)	(0.52)
Women								
Age 25	27.2	27.1	33.7	33.7	36.9	37.0	39.6	39.7
	(1.58)	(1.25)	(1.53)	(1.23)	(1.39)	(1.04)	(1.32)	(1.05)
Age 35	22.6	22.5	27.9	27.9	30.6	30.7	32.6	32.7
	(1.25)	(0.98)	(1.20)	(0.96)	(1.09)	(0.81)	(1.02)	(0.80)
Age 45	18.0	18.0	22.2	22.2	24.3	24.4	25.7	25.8
	(0.97)	(0.75)	(0.91)	(0.73)	(0.82)	(0.61)	(0.76)	(0.58)
Age 55	13.7	13.6	16.8	16.8	18.4	18.4	19.3	19.3
	(0.72)	(0.56)	(0.66)	(0.52)	(0.59)	(0.44)	(0.53)	(0.40)

Standard errors are shown in brackets.

9. Comparison with estimates obtained when excluding patients without ID

Excluding patients without recorded IDs from the analysis reduces the number of patients from 37,740 to 30,287. Because the analysis is limited only to patients whose vital status can be established through the national population register, it is not necessary to make any assumptions about mortality in patients who are lost to follow-up, or to apply inverse probability weighting. Analysis closure is defined to be the date 30 days prior to the date at which the vital status of the patient is last checked against the population register (the interval of 30 days was included to allow for potential delays in the reporting of deaths). This is later than the analysis closure date calculated if follow-up after 6 months prior to the last visit is excluded (the approach that is used in the main analysis for the purpose of identifying LTFU cases). As a result, the average follow-up time in this subset of patients (2.75 years) is longer than that in the main analysis (1.84 years).

Table 4 shows the estimates of the parameters in the model. Mortality after the first 12 months of ART was found to be significantly lower in men starting ART after 2006 than in men starting ART up to 2006 (HR 0.69, 95% CI: 0.55-0.87) and lower in women starting ART after 2006 than in women starting ART up to 2006, though the female difference was only of borderline significance (HR 0.83, 95% CI: 0.67-1.02). Life expectancies were therefore calculated separately for patients with IDs starting ART up to 2006 and after 2006, and compared with the estimates from the main analysis (Table 5). For male patients with

IDs, starting ART up to 2006, life expectancies were substantially higher than those in the main analysis – by 15-20% in patients with baseline CD4 values <50 cells/µl and by about 10% in other CD4 categories. This is likely to be due to the longer follow-up in the sensitivity analysis, which led to a relatively low estimate of relative mortality at durations >36 months (HR 0.28 in Table 4 compared to HR 0.44 in Table 1). For female patients with IDs, starting ART up to 2006, life expectancies were similar to those in the main analysis. Standard errors were smaller in the sensitivity analysis than in the main analysis, due to the greater number of person years at the longer follow-up durations.

Life expectancies in patients with recorded ID were higher when comparing those starting ART after 2006 to those starting ART up to 2006. The relative difference was greatest in those patients starting ART at low CD4 counts and at younger ages.

Table 4: Mortality rates and model estimates when analysis is restricted to patients with recorded ID numbers

	Symbol	Males, by time since ART start		Females, by time since ART start	
		<12 month	>12 months	<12 month	>12 months
Number of deaths		1,707	760	1,970	774
Person years		10,445	20,969	16,481	35,304
Crude mortality rate per PYO		0.1634	0.0362	0.1195	0.0219
Excess HIV mortality	$\exp(\gamma_d)$	0.109 (0.079-0.150)	0.024 (0.012-0.046)	0.096 (0.075-0.123)	0.019 (0.012-0.032)
Relative HIV mortality at					
Durations 25-36 months	$\exp(\gamma_2 - \gamma_1)$	-	0.62 (0.50-0.78)	-	0.56 (0.45-0.69)
Durations >36 months	$\exp(\gamma_3 - \gamma_1)$	-	0.28 (0.20-0.40)	-	0.40 (0.30-0.52)
Increase in HIV mortality for each 10-year increase in age	$\exp(10\eta_d)$	1.11 (1.04-1.17)	1.03 (0.91-1.15)	1.10 (1.05-1.16)	1.04 (0.93-1.16)
Hazard ratio relative to CD4 <50					
Baseline CD4 50-99	$\exp(g_d(1))$	0.57 (0.50-0.65)	0.81 (0.62-1.05)	0.48 (0.42-0.54)	0.75 (0.60-0.94)
Baseline CD4 100-199	$\exp(g_d(2))$	0.31 (0.27-0.35)	0.61 (0.48-0.77)	0.25 (0.22-0.28)	0.51 (0.41-0.63)
Baseline CD4 200+	$\exp(g_d(3))$	0.20 (0.16-0.25)	0.59 (0.42-0.83)	0.22 (0.19-0.27)	0.38 (0.27-0.54)
Hazard ratio relative to cohort 4					
Cohort 1	$\exp(\theta_d(1))$	1.93 (1.48-2.51)	3.36 (1.97-5.73)	1.93 (1.56-2.38)	2.53 (1.75-3.66)
Cohort 2	$\exp(\theta_d(2))$	3.03 (2.36-3.88)	2.49 (1.53-4.54)	3.42 (2.82-4.16)	2.70 (1.89-3.86)
Cohort 3	$\exp(\theta_d(3))$	1.90 (1.45-2.49)	2.63 (0.70-1.50)	1.45 (0.86-2.44)	2.20 (0.93-5.21)
Cohort 5	$\exp(\theta_d(5))$	1.01 (0.77-1.33)	1.38 (0.78-2.43)	1.04 (0.84-1.29)	1.47 (1.00-2.17)
Cohort 6	$\exp(\theta_d(6))$	1.29 (0.92-1.82)	0.50 (0.18-1.37)	1.57 (1.20-2.07)	0.27 (0.09-0.79)
Relative HIV mortality if starting ART after 2006	-	0.92 (0.82-1.03)	0.69 (0.55-0.87)	0.92 (0.84-1.02)	0.83 (0.67-1.02)

95% confidence intervals are shown in brackets. PYO = person-year of observation.

Table 5: Comparison of life expectancy estimates using all patient records and only records of patients with IDs (by year of ART initiation)

	Baseline CD4 <50			Baseline CD4 50-99			Baseline CD4 100-199			Baseline CD4 200+		
	All	ID patients		All	ID patients		All	ID patients		All	ID patients	
	patients	≤2006	>2006	patients	≤2006	>2006	patients	≤2006	>2006	patients	≤2006	>2006
Men												
Age 25	19.8	23.8	26.3	25.0	27.5	29.8	28.1	30.9	32.9	28.7	31.8	33.8
	(1.20)	(1.02)	(1.07)	(1.30)	(1.09)	(1.05)	(1.25)	(0.90)	(0.86)	(1.56)	(1.16)	(1.05)
Age 35	16.3	19.5	21.4	20.5	22.7	24.4	23.1	25.4	26.9	23.6	26.2	27.6
	(0.92)	(0.73)	(0.76)	(0.99)	(0.78)	(0.75)	(0.95)	(0.64)	(0.61)	(1.19)	(0.83)	(0.75)
Age 45	12.7	15.1	16.4	16.0	17.6	18.8	18.0	19.8	20.7	18.4	20.4	21.3
	(0.71)	(0.54)	(0.54)	(0.74)	(0.56)	(0.52)	(0.70)	(0.45)	(0.42)	(0.88)	(0.57)	(0.51)
Age 55	9.5	11.0	11.9	11.8	12.9	13.7	13.3	14.5	15.1	13.7	15.0	15.6
	(0.53)	(0.41)	(0.39)	(0.53)	(0.40)	(0.36)	(0.50)	(0.32)	(0.28)	(0.61)	(0.38)	(0.33)
Women												
Age 25	27.2	27.9	29.7	33.7	33.2	34.9	36.9	37.7	39.0	39.6	39.6	40.7
	(1.58)	(1.24)	(1.34)	(1.53)	(1.29)	(1.32)	(1.39)	(1.03)	(1.01)	(1.32)	(1.08)	(1.04)
Age 35	22.6	23.2	24.7	27.9	27.7	29.0	30.6	31.3	32.3	32.6	32.8	33.6
	(1.25)	(1.00)	(1.05)	(1.20)	(1.03)	(1.03)	(1.09)	(0.81)	(0.77)	(1.02)	(0.82)	(0.78)
Age 45	18.0	18.5	19.6	22.2	22.2	23.1	24.3	24.9	25.6	25.7	25.9	26.5
	(0.97)	(0.81)	(0.82)	(0.91)	(0.80)	(0.77)	(0.82)	(0.63)	(0.58)	(0.76)	(0.60)	(0.57)
Age 55	13.7	14.0	14.8	16.8	16.8	17.4	18.4	18.8	19.3	19.3	19.5	19.9
	(0.72)	(0.63)	(0.62)	(0.66)	(0.59)	(0.56)	(0.59)	(0.46)	(0.41)	(0.53)	(0.42)	(0.39)

Standard errors are shown in brackets.

10. Comparison with life expectancy estimates 24 months after starting ART

Table 6 shows that the life expectancies calculated for individuals who have survived 24 months after starting ART are substantially greater than the life expectancies of individuals of the same age who have just started therapy. This difference is a reflection of the high mortality risk that exists during the first two years after starting ART, and is proportionally greatest in individuals starting ART with CD4 counts <50 cells/µl.

Table 6: Comparison of life expectancy estimates at ART initiation and 24 months after ART initiation

	CD4 <50		CD4 50-99		CD4 100-199		CD4 200+	
	ART start	24 months	ART start	24 months	ART start	24 months	ART start	24 months
Men								
Age 25	19.8	25.2	25.0	28.6	28.1	32.8	28.7	35.5
	(1.20)	(1.67)	(1.30)	(1.59)	(1.25)	(1.44)	(1.56)	(1.36)
Age 35	16.3	21.0	20.5	23.7	23.1	26.9	23.6	28.8
	(0.92)	(1.32)	(0.99)	(1.24)	(0.95)	(1.09)	(1.19)	(1.01)
Age 45	12.7	16.8	16.0	18.7	18.0	20.9	18.4	22.2
	(0.71)	(1.03)	(0.74)	(0.93)	(0.70)	(0.78)	(0.88)	(0.71)
Age 55	9.5	12.7	11.8	14.0	13.3	15.4	13.7	16.2
	(0.53)	(0.77)	(0.53)	(0.67)	(0.50)	(0.53)	(0.61)	(0.46)
Women								
Age 25	27.2	33.9	33.7	37.4	36.9	41.4	39.6	45.4
	(1.58)	(1.72)	(1.53)	(1.47)	(1.39)	(1.13)	(1.32)	(0.69)
Age 35	22.6	28.5	27.9	31.3	30.6	34.2	32.6	37.1
	(1.25)	(1.37)	(1.20)	(1.15)	(1.09)	(0.87)	(1.02)	(0.52)
Age 45	18.0	23.1	22.2	25.0	24.3	27.1	25.7	29.1
	(0.97)	(1.06)	(0.91)	(0.87)	(0.82)	(0.64)	(0.76)	(0.37)
Age 55	13.7	17.8	16.8	19.1	18.4	20.4	19.3	21.7
	(0.72)	(0.77)	(0.66)	(0.62)	(0.59)	(0.44)	(0.53)	(0.25)

11. Comparison with alternative non-HIV mortality assumptions

The model was refitted after increasing the assumed non-HIV mortality rates to levels 50% above those estimated in the ASSA2008 model. This led to a reduction in life expectancy in all age and CD4 strata (Table 7). The reduction was proportionally greatest in older individuals, in women and in individuals starting ART at higher CD4 counts. As shown in Figure 2 of the main text, these are likely to be the groups in which non-HIV mortality accounts for a relatively high proportion of deaths.

Table 7: Comparison of life expectancy estimates using different non-HIV mortality assumptions

	CD4 <50		CD4 50-99		CD4 100-199		CD4 200+	
	Base non-HIV rates	Base × 1.5	Base non-HIV rates	Base × 1.5	Base non-HIV rates	Base × 1.5	Base non-HIV rates	Base × 1.5
Men								
Age 25	19.8 (1.20)	19.3 (1.11)	25.0 (1.30)	24.1 (1.13)	28.1 (1.25)	27.0 (1.00)	28.7 (1.56)	27.6 (1.26)
Age 35	16.3 (0.92)	15.8 (0.78)	20.5 (0.99)	19.6 (0.80)	23.1 (0.95)	21.9 (0.69)	23.6 (1.19)	22.5 (0.89)
Age 45	12.7 (0.71)	12.2 (0.55)	16.0 (0.74)	14.9 (0.54)	18.0 (0.70)	16.6 (0.46)	18.4 (0.88)	17.1 (0.58)
Age 55	9.5 (0.53)	8.8 (0.38)	11.8 (0.53)	10.6 (0.35)	13.3 (0.50)	11.8 (0.29)	13.7 (0.61)	12.2 (0.36)
Women								
Age 25	27.2 (1.58)	26.4 (1.35)	33.7 (1.53)	32.2 (1.27)	36.9 (1.39)	35.0 (1.12)	39.6 (1.32)	37.2 (0.99)
Age 35	22.6 (1.25)	21.7 (0.98)	27.9 (1.20)	26.3 (0.91)	30.6 (1.09)	28.5 (0.80)	32.6 (1.02)	30.1 (0.70)
Age 45	18.0 (0.97)	16.9 (0.70)	22.2 (0.91)	20.4 (0.63)	24.3 (0.82)	22.1 (0.55)	25.7 (0.76)	23.1 (0.47)
Age 55	13.7 (0.72)	12.4 (0.48)	16.8 (0.66)	14.9 (0.41)	18.4 (0.59)	16.1 (0.35)	19.3 (0.53)	16.7 (0.30)

12. Comparison with negative binomial model

We tested the validity of the Poisson assumption using the test for over-dispersion recommended by Dean and Lawless [35]. Although we found no evidence against the Poisson assumption when restricting the analysis to patients with IDs, we found that in the main analysis, where we used inverse probability weighting to increase the weight assigned to individuals with IDs who were lost to follow-up, there was evidence of over-dispersion, both in males ($p < 0.001$) and in females ($p < 0.001$). We therefore performed a sensitivity analysis to assess whether the results changed when using a negative binomial model, which is more appropriate in cases where there is significant over-dispersion. Average life expectancy estimates were almost identical to those obtained using the Poisson model, although standard error estimates tended to be slightly higher when using the negative binomial model (Table 8).

Table 8: Comparison of life expectancy estimates obtained using a Poisson model (PM) and a negative binomial model (NBM)

	CD4 <50		CD4 50-99		CD4 100-199		CD4 200+	
	PM	NBM	PM	NBM	PM	NBM	PM	NBM
Men								
Age 25	19.8	19.9	25.0	25.1	28.1	27.9	28.7	28.5
	(1.20)	(1.29)	(1.30)	(1.47)	(1.25)	(1.29)	(1.56)	(1.69)
Age 35	16.3	16.4	20.5	20.6	23.1	22.9	23.6	23.5
	(0.92)	(0.98)	(0.99)	(1.11)	(0.95)	(0.97)	(1.19)	(1.27)
Age 45	12.7	12.8	16.0	16.0	18.0	17.9	18.4	18.4
	(0.71)	(0.75)	(0.74)	(0.81)	(0.70)	(0.72)	(0.88)	(0.93)
Age 55	9.5	9.5	11.8	11.9	13.3	13.2	13.7	13.7
	(0.53)	(0.56)	(0.53)	(0.57)	(0.50)	(0.51)	(0.61)	(0.64)
Women								
Age 25	27.2	27.3	33.7	33.6	36.9	36.3	39.6	39.6
	(1.58)	(1.68)	(1.53)	(1.67)	(1.39)	(1.55)	(1.32)	(1.46)
Age 35	22.6	22.5	27.9	27.8	30.6	30.1	32.6	32.5
	(1.25)	(1.33)	(1.20)	(1.31)	(1.09)	(1.23)	(1.02)	(1.14)
Age 45	18.0	17.8	22.2	22.0	24.3	23.9	25.7	25.6
	(0.97)	(1.03)	(0.91)	(1.00)	(0.82)	(0.94)	(0.76)	(0.85)
Age 55	13.7	13.4	16.8	16.6	18.4	18.0	19.3	19.2
	(0.72)	(0.77)	(0.66)	(0.73)	(0.59)	(0.68)	(0.53)	(0.60)

Standard errors are shown in brackets.

References

1. Dickman PW, Sloggett A, Hills M, Hakulinen T (2004) Regression models for relative survival. Stat Med 23: 51-64.
2. Ederer F, Axtell LM, Cutler SJ (1961) The relative survival rate: a statistical methodology. Natl Cancer Inst Monogr 6: 101-121.
3. van Sighem AI, Gras LA, Reiss P, Brinkman K, de Wolf F (2010) Life expectancy of recently diagnosed asymptomatic HIV-infected patients approaches that of uninfected individuals. AIDS 24: 1527-1535.
4. van Sighem A, Danner S, Ghani AC, Gras L, Anderson RM, et al. (2005) Mortality in patients with successful initial response to highly active antiretroviral therapy is still higher than in non-HIV-infected individuals. J Acquir Immun Defic Syndr 40: 212-218.
5. Bhaskaran K, Hamouda O, Sannes M, Boufassa F, Johnson AM, et al. (2008) Changes in the risk of death after HIV seroconversion compared with mortality in the general population. JAMA 300: 51-59.
6. Brinkhof MW, Boulle A, Weigel R, Messou E, Mathers C, et al. (2009) Mortality of HIV-infected patients starting antiretroviral therapy in sub-Saharan Africa: comparison with HIV-unrelated mortality. PLoS Med 6: e1000066.
7. Cornell M, Grimsrud A, Fairall L, Fox MP, van Cutsem G, et al. (2010) Temporal changes in programme outcomes among adult patients initiating antiretroviral therapy across South Africa, 2002-2007. AIDS 24: 2263-2270.
8. Boulle A, Van Cutsem G, Hilderbrand K, Cragg C, Abrahams M, et al. (2010) Seven year experience of a primary care antiretroviral treatment programme in Khayelitsha, South Africa. AIDS 24: 563-572.
9. Nglazi MD, Lawn SD, Kaplan R, Kranzer K, Orrell C, et al. (2011) Changes in programmatic outcomes during 7 years of scale-up at a community-based antiretroviral treatment service in South Africa. J Acquir Immun Defic Syndr 56: e1-8.
10. Fatti G, Grimwood A, Bock P (2010) Better antiretroviral therapy outcomes at primary healthcare facilities: an evaluation of three tiers of ART services in four South African provinces. PLoS One 5: e12888.
11. Mutevedzi PC, Lessells RJ, Rodger AJ, Newell ML (2011) Association of age with mortality and virological and immunological response to antiretroviral therapy in rural South African adults. PLoS One 6: e21795.
12. Micheloud D, Berenguer J, Bellón JM, Miralles P, Cosin J, et al. (2008) Negative influence of age on CD4+ cell recovery after highly active antiretroviral therapy in naive HIV-1-infected patients with severe immunodeficiency. J Infect 56: 130-136.
13. Sabin CA, Smith CJ, d'Arminio Monforte A, Battegay M, Gabiano C, et al. (2008) Response to combination antiretroviral therapy: variation by age. AIDS 22: 1463-1473.
14. Nash D, Katyal M, Brinkhof MW, Keiser O, May M, et al. (2008) Long-term immunologic response to antiretroviral therapy in low-income countries: a collaborative analysis of prospective studies. AIDS 22: 2291-2302.
15. Vella V, Govender T, Dlamini S, Taylor M, Moodley I, et al. (2010) Retrospective study on the critical factors for retaining patients on antiretroviral therapy in KwaZulu-Natal, South Africa. J Acquir Immun Defic Syndr 55: 109-116.

16. Setel PW, Macfarlane SB, Szreter S, Mikkelsen L, Jha P, et al. (2007) A scandal of invisibility: making everyone count by counting everyone. Lancet 370: 1569-1577.
17. Murray CJL, Ferguson BD, Lopez AD, Guillot M, Salomon JA, et al. (2003) Modified logit life table system: principles, empirical validation, and application. Pop Studies 57: 165-182.
18. Brass W (1971) On the scale of mortality. In: Brass W, editor. Biological Aspects of Demography. London: Taylor & Francis.
19. Actuarial Society of South Africa (2011) ASSA2008 AIDS and Demographic Model. Available: http://aids.actuarialsociety.org.za. Accessed 5 April 2011.
20. Dorrington R, Bradshaw D, Wegner T (1999) Estimates of the Level and Shape of Mortality Rates in South Africa around 1985 and 1990 Derived by Applying Indirect Demographic Techniques to Reported Deaths. Cape Town: Medical Research Council, Centre for Epidemiological Research in South Africa. Available: http://www.mrc.ac.za/bod/1999report.pdf. Accessed 1 Feb 2012.
21. Dusheiko GM, Brink BA, Conradie JD, Marimuthu T, Sher R (1989) Regional prevalence of hepatitis B, delta, and human immunodeficiency virus infection in southern Africa: a large population survey. Am J Epidemiol 129: 138-145.
22. Schoub BD, Lyons SF, McGillivray GM, Smith AN, Johnson S, et al. (1987) Absence of HIV infection in prostitutes and women attending sexually-transmitted disease clinics in South Africa. Trans R Soc Trop Med Hyg 81: 874-875.
23. Statistics South Africa (2010) Mortality and causes of death in South Africa, 2008: Findings from death notification. Pretoria. Available: http://www.statssa.gov.za/publications/statsdownload.asp?PPN=P0309.3&SCH=4790 . Accessed 28 Nov 2010.
24. Johnson LF, Hallett TB, Rehle TM, Dorrington RE (2012) The effect of changes in condom usage and antiretroviral treatment coverage on HIV incidence in South Africa: a model-based analysis. J Roy Soc Interface 9: 1544-1554.
25. Groenewald P, Nannan N, Bourne D, Laubscher R, Bradshaw D (2005) Identifying deaths from AIDS in South Africa. AIDS 19: 193-201.
26. Birnbaum JK, Murray CJ, Lozano R (2011) Exposing misclassified HIV/AIDS deaths in South Africa. Bull WHO 89: 278-285.
27. Cornell M, Technau K, Fairall L, Wood R, Moultrie H, et al. (2009) Monitoring the South African antiretroviral programme 2003-2007: the IeDEA Southern Africa Collaboration. S Afr Med J 99: 653-660.
28. Adam MA, Johnson LF (2009) Estimation of adult antiretroviral treatment coverage in South Africa. S Afr Med J 99: 661-667.
29. Gregson S, Garnett GP (2000) Contrasting gender differentials in HIV-1 prevalence and associated mortality increase in eastern and southern Africa: artefact of data or natural course of epidemics? AIDS 14 (Suppl 3): S85-99.
30. Blacker J, Zaba B (1997) HIV prevalence and lifetime risk of dying of AIDS. Health Trans Rev 7 (Suppl 2): 45-62.
31. Wambura M, Urassa M, Isingo R, Ndege M, Marston M, et al. (2007) HIV prevalence and incidence in rural Tanzania: results from 10 years of follow-up in an open-cohort study. J Acquir Immun Defic Syndr 46: 616-623.
32. Chiang CL (1968) The life table and its construction. Introduction to Stochastic Processes in Biostatistics. New York: John Wiley & Sons. pp. 189-217.
33. Chiang CL (1972) On constructing current life tables. J Am Stat Assoc 67: 538-541.
34. van Buuren S, Boshuizen HC, Knook DL (1999) Multiple imputation of missing blood pressure covariates in survival analysis. Stat Med 18: 681-694.

35. Dean C, Lawless JF (1989) Tests for detecting overdispersion in Poisson regression models. J Am Stat Assoc 84: 467-472.
